# On the Factors Causing Processing Difficulty of Multiple-Scene Displays

**DOI:** 10.1177/2041669516689572

**Published:** 2017-03-08

**Authors:** Matthew J Stainer, Kenneth C Scott–Brown, Benjamin W Tatler

**Affiliations:** School of Psychology, University of Aberdeen, UK; Division of Psychology, Abertay University, UK; School of Psychology, University of Aberdeen, UK

**Keywords:** Multiple-scene viewing, multiplex displays, change detection, scene viewing, attention

## Abstract

Multiplex viewing of static or dynamic scenes is an increasing feature of screen media. Most existing multiplex experiments have examined detection across increasing scene numbers, but currently no systematic evaluation of the factors that might produce difficulty in processing multiplexes exists. Across five experiments we provide such an evaluation. Experiment 1 characterises difficulty in change detection when the number of scenes is increased. Experiment 2 reveals that the increased difficulty across multiple-scene displays is caused by the total amount of visual information accounts for differences in change detection times, regardless of whether this information is presented across multiple scenes, or contained in one scene. Experiment 3 shows that whether quadrants of a display were drawn from the same, or different scenes did not affect change detection performance. Experiment 4 demonstrates that knowing which scene the change will occur in means participants can perform at monoplex level. Finally, Experiment 5 finds that changes of central interest in multiplexed scenes are detected far easier than marginal interest changes to such an extent that a centrally interesting object removal in nine screens is detected more rapidly than a marginally interesting object removal in four screens. Processing multiple-screen displays therefore seems dependent on the amount of information, and the importance of that information to the task, rather than simply the number of scenes in the display. We discuss the theoretical and applied implications of these findings.

## Introduction

The spatial and temporal constraints on human attention allocation in single scenes have been described in great detail (see Tatler, 2009). However, we are commonly presented with information that traverses multiple displays, such as multiple monitor computer setups, multi-scene television viewing (e.g. some news channels), and banks of monitors in CCTV control rooms (Stainer, Scott-Brown, & Tatler, 2013b). Any potential benefit of maximising the amount of visual information in this way must depend crucially on the limits of the human observer in processing this information (Attwood & Effron, 2006). While these multi-scene displays have permeated the way we interact with media, little is understood about how theory of scene perception generated from single scene viewing applies in these multiplex viewing conditions which not only present the visual system with an increased visual load, but also introduce further potential sources of processing difficulty - such as the need to manage attention conflicts not only within a scene, but also between the scenes in a display.

The factors that underlie the allocation of gaze when looking at multiplexed scenes may not be the same as found in single scene viewing, such as the frame of reference around which inspection is organised during a multiplex scene-memorisation task (Stainer, Scott-Brown, & Tatler, 2013a). Moreover, when given the choice, expert CCTV operators view content on their single scene spot-monitors rather than the multiplexed wall of monitors, suggesting both a reliance on prior knowledge to guide their search for crime, and an understanding of the difficulty of processing such large data loads (Stainer et al., 2013b). Research into processing multiple scene displays has tended to focus on the decrease in detection performance associated with multiplex viewing (e.g. Tickner & Poulton, 1973), however, as of yet a systematic evaluation of the factors that might influence difficulties in multiplex viewing has not been conducted. We used a change detection task to explore the contribution of several potential sources of processing difficulty in multiple-scene displays based on previously observed relationships between items in a scene, or an array of scenes.

### Increased information content

Set-size effects demonstrate that the number of distractor items in an array correlates very strongly with search latencies (Wolfe, 1998). Correspondingly, the time to detect changes to arrays increases when the number of items increases (Bravo & Farid, 2004; Rensink, 2000; Wilken & Ma, 2004; Wright, Green, & Baker, 2000). Beck, Levin, and Angelone (2007) and Zelinsky (2001) have further demonstrated that change detection in natural scenes is related to the number of objects within the scene, however, the number of objects in each of these experiments was relatively easy to distinguish (for example, there was little overlap between objects), whereas segmenting natural scenes is not always such a simple process. Yokosawa and Mitsumatsu (2003) revealed that when only a certain number of segments of a visual image was shown in a change detection task, change detection performance scaled with the amount of visual information. Research has begun to address alternative ways to quantify the amount of visual information that is contained in real-world scenes (Asher, Tolhurst, Troscianko, & Gilchrist, 2013; Bravo & Farid, 2004, 2008; Henderson, Chanceaux, & Smith, 2009; Neider & Zelinsky, 2008, 2010; Rosenholtz, Li, Mansfield, & Jin, 2005; Rosenholtz, Li, & Nakano, 2007). Regardless of how visual information is quantified, the overwhelming message is that a larger amount of measured visual information tends to correlate with longer search times.

### Physical extent of the display

Given acuity limits in the peripheral retina, increasing the physical extent of a search array is likely to influence search performance. This is demonstrated in the well-documented effects of eccentricity in visual search (Carrasco & Chang, 1995; Carrasco & Yeshurun, 1998; Wolfe, O'Neill, & Bennett, 1998). In complex scenes, the physical extent of the scene on the display changes viewing behaviour: while larger scenes necessarily result in larger saccades, when saccade sizes are expressed relative to the size of the scene, larger scenes result in relatively smaller saccade amplitudes (von Wartburg et al., 2007). However, physical size effects may be more complex than larger displays simply resulting in worse search performance. Ball and North (2005) partially separated the influence of display size from that of the visual content of a scene by comparing search of a scene displayed over a multiplex of 1, 4 and 9 displays. The search array was initially scaled to each display size, with participants able to pan and zoom in the images (to effectively make the targets the same size on the monoplex as the nonaplex). The authors noted, however, that participants preferred not to use these controls, and were more likely to physically navigate the images in zoomed out mode. Search performance for small targets was better for the 9-screen multiplex (nonaplex) arrays than for the 4-screen (quadraplex) or single screen (monoplex) displays. They argued that this might be due to the larger display allowing a higher level of detail of small targets.

### Physical discontinuity

An additional consequence of presenting information across a multiplex display is that content is often separated by physical space, either by the bezels of a monitor, or by artificial windowing. In Varakin and Levin (2008), when a scene was simply segmented into four sections by windows, detection performance was unaffected by this artificial windowing. Similar patterns of results have also been found in visual search tasks across multiple monitors (Bi, Bae, & Balakrishnan, 2010; Hutchings, Czerwinski, & Meyers, 2004; Tan & Czerwinski, 2003), suggesting that search for targets is unaffected by the presence or absence of a physical separation, or boundaries between content. When boundaries are introduced between the four quadrants of a scene, fixation selection is only significantly changed when the order of the quadrants are also scrambled (Stainer et al., 2013a). Thus, the mere presence of boundaries may not influence attention, but only when coherent scene structure can be perceptually bridged across the gaps.

### Semantic discontinuity

Visual and semantic similarity between items influences search performance. In terms of visual similarity, targets become harder to detect as target-distractor similarity increases (J. Beck & Ambler, 1973; Treisman & Gelade, 1980), or as variance in the distractor items increases (Duncan & Humphreys, 1989; Smith, Pickett, & Trutschl, 1998). Semantic similarity between target and distractors can also influence search performance. When searching for a target presented with distractors from the same category (e.g. the number 4 presented among other numbers), search times are longer than when searching for the same target among distractors from another category (e.g. the number 4 among letters; see Jonides & Gleitman, 1972). These effects of semantic category can be shown to arise from endogenous understanding of the display rather than purely physical visual properties: Jonides and Gleitman (1972) used the physically identical symbol O, but varied whether they told participants to search for the digit zero or the letter o. In this situation the same between-category search benefits were shown depending upon the instructions given to the participants. However, subsequent studies failed to replicate this finding (Duncan, 1983), and it was proposed that category effects disappear when items are matched in physical properties (Krueger, 1984). More recently, Lupyan (2008) varied the conceptual but not visual variability between distractors in a search task. Targets (letter-like shapes) were easier to find when the distractors were conceptually homogenous (distractors were B and b, thus sharing the conceptual representation of the same letter) than when distractors were conceptually heterogeneous (e.g. B and p as distractors, thus representing different letter concepts). This effect was even stronger when participants were asked to verbally label the categories. It seems consistent that categorising objects in a search task (either in a perceptual or conceptual manner), improves both search times and efficiency (Wolfe, Friedman-Hill, Stewart, & O'Connell, 1992).

Luck and Vogel (1997) suggested that as the number of items in an array increases, it is likely that as each item introduces an amount of visual information load associated with the object. If a new item is semantically similar to those already in the display, then each object should be associated with a lesser demand on working memory than objects that are different. Alvarez and Cavanaugh (2004) found that both the number of objects, and the visual information load associated with those objects influenced change detection. However, Barton, Ester, and Awh (2009) found that the variance between items in a change detection task (e.g. the display was either made up of cubes, or Chinese characters, or a mix of both) did not influence peoples ability to detect changes. In multiple-scene displays, competition for attention does not only exist between the items within a scene, but also between the different scenes in the multiplex. Greene and Wolfe (2011) asked participants to determine whether scenes with a particular global property were present or absent in displays containing arrays of one to four scenes. They found that search was inefficient when there was more than one scene on the display. Thus, it is unclear if semantic information at a scene level (which is inherently a global property of each scene: it is the sum of the information contained in the scene) would be processed when attending to the multiplex, or whether the multiplex itself is treated as one global semantic unit containing local variance in content (i.e. between scenes) much as is true of the local variance within single scenes.

### Viewing multiple scenes

Although multiplex displays present these challenges for human perception, to date, surprisingly little research has considered how we attend to multiplex displays. Notable exceptions include studies of rapid processing of briefly presented multiplex displays. Rousselet, Thorpe, and Fabre–Thorpe (2004) studied animal detection performance in briefly presented scenes. These authors found that increasing the number of scenes presented simultaneously from 1 to 2 to 4 scenes resulted in increases in reaction time. Behavioural costs when faced with increasing numbers of scenes had been previously demonstrated by Tickner and Poulton (1973) using a surveillance-based task (also see Howard, Gilchrist, Troscianko, Behera, & Hogg, 2011). These authors showed that when monitoring simultaneous feeds from cameras in a prison, the accuracy with which participants detected suspicious events was lower when the number of simultaneously-viewed camera feeds was high; with 83% for 4 monitors, 84% for 9 monitors and 64% for 16 monitors. Similarly, Wallace, Diffley, Baines, and Aldridge (1997) examined participants target detection across multiple scenes and found decreases in performance when increasing the number of scenes presented in the town centre CCTV array.

### The present study

In this series of experiments, we use change detection to explore processing of multiple scene displays. The flicker paradigm is, as has been shown (e.g. in Rensink, O'Regan, & Clark, 1997), a convenient laboratory paradigm for studying how attention is allocated to complex visual scenes, with successful detection of a change being contingent upon the allocation of attention to the site of the change (Cohen, Cavanagh, Chun, & Nakayama, 2012; Henderson & Hollingworth, 1999), which is not necessarily guaranteed upon fixation. Thus the time taken to locate a change reflects how long it took participants to attend to the location of the change. Change detection has the further advantage in the study of attention allocation in that the target does not need to be defined before the trial, therefore reducing the utility of contextual guidance that can inform visual search tasks for a target which has a learned spatial relationship with an environment (e.g. clocks tend to be on walls, not ceilings; Torralba, Oliva, Castelhano, & Henderson, 2006). Such contextual cueing can serve to reduce the area over which an observer must search for a target (Zelinsky & Schmidt, 2009). Providing no prior information about the identity of the changed item means participants could expect the target to be in any location in the scene or multiplex.

We used the flicker paradigm with trials in which a single changed item in one of the scenes was presented in a display amongst several unchanging scenes. In Experiment 1 we assessed how change detection is affected by increasing the number of scenes displayed simultaneously from 1 to 4 to 9 scenes (monoplex, quadraplex and nonaplex respectively). This experiment allowed us to characterise performance decreases across our multiplexes. In Experiment 2 and 3 we tease apart the potential sources of this processing difficulty. In Experiment 4, we ask whether knowing about the scene containing the change alters the task difficulty. Finally, Experiment 5 examines how semantic importance of the changed item affects multiplex change detection difficulty.

## Experiment 1

Experiment 1 examined change detection performance in monoplex, quadraplex and nonaplex displays to characterise performance difficulty in our task. Attentional allocation was indexed by considering the latency and accuracy of change detection performance using a survival analysis technique (explained in methods below). Given previous literature on multi-scene viewing (Tickner & Poulton, 1973; Wallace & Diffley, 1998), increasing the number of scenes displayed simultaneously will result in longer latencies for change detection.

### Methods

#### Participants

Fifteen undergraduate psychology students took part in Experiment 1 in exchange for course credit. Participants in all studies reported here reported normal or corrected-to-normal vision. Participants provided written consent to take part in each of the experiments presented in this paper. The research was approved by the University of Dundee Human Research Ethics Committee, and conducted in accordance with the Declaration of Helsinki.

#### Stimuli

**Figure 1.**
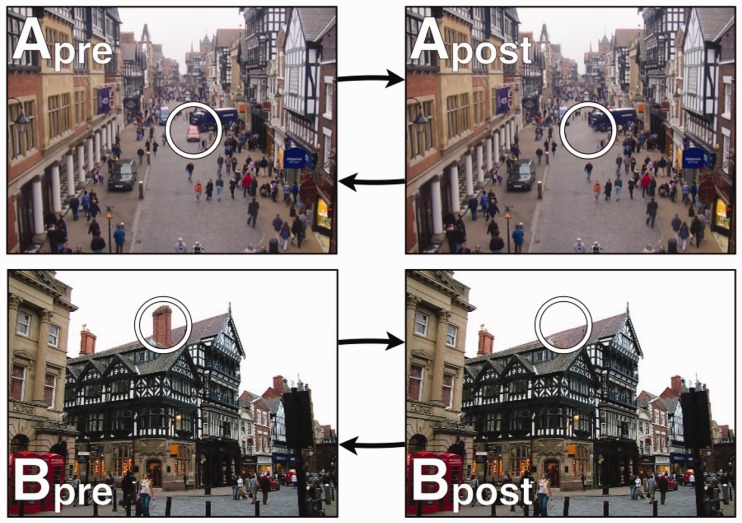
Two representative example scenes in pre and post-change format. The changed items in each scene is indicated by the white circle. (A) A disappearing van, (B) a disappearing chimney.

126 digital colour photographs of city centre scenes were used for Experiment 1. The photographs were 400 × 300 pixels in size (those that were above were cropped or scaled to this size). Of these, 27 photographs were randomly selected to be target images where changes were made to a single item using Adobe Photoshop. These items were removed using the ‘cloning’ tool, where the changed item was replaced with appropriate estimated background texture. This technique makes it possible to change items that are overlapping, such as digitally removing a person standing in front of a shop window. Examples of items that were digitally removed include windows, people, chimneys, road markings, shop signs and cars (see Figure 1). Changes were designed to ensure that both the pre- and post-changed scene were physically plausible (for example a lamp-post would not be removed, leaving a gravity-defying floating light). The changes were to items that were definable as discrete objects and care was taken to ensure that image or editing flaws did not reveal the location of the change. Accordingly, correct detection of change would require the observer to have inspected both pre- and post-change versions of the stimulus image rather than by them perceiving digital image flaw ‘pop-out’.

**Figure 2.**
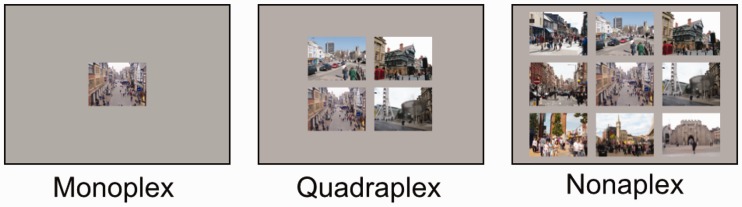
Monoplex, quadraplex and nonaplex displays of a single pre-changed scene in each of the three conditions. The target scene appears in the bottom left of quadraplex and in the centre of the nonaplex display in this figure. During the experiment, the location of the changing scene in the multiplex arrays was randomised.

Stimuli were presented in monoplex, quadraplex or nonaplex displays (see Figure 2 for sample arrangements). Displays were presented in a random order, and were counterbalanced so that the changed photograph could exist in any position of the four, or nine possible display positions. Stimuli were piloted [n=5] to ensure that when presented in monoplex format, average detection was made in fewer than 10 alternations.

#### Apparatus

Stimuli were presented on a 24inch iMac 3.06GHz Intel Core 2 Duo with 4GB 1067 MHz DDR2 SDRAM operating with a screen resolution of 1920 × 1200. The experiment was programmed and run using MatLab version 7.8.0, using PsychToolbox (Brainard, 1997; Pelli, 1997). Viewing was conducted at a viewing distance of approximately 60 cm, with the monitor subtending an approximate 40 degree visual angle horizontally. The size of a single scene was 400 × 300 pixels, the inter-scene distance for multiplex arrays was 50 pixels vertically and horizontally.

#### Procedure

**Figure 3.**
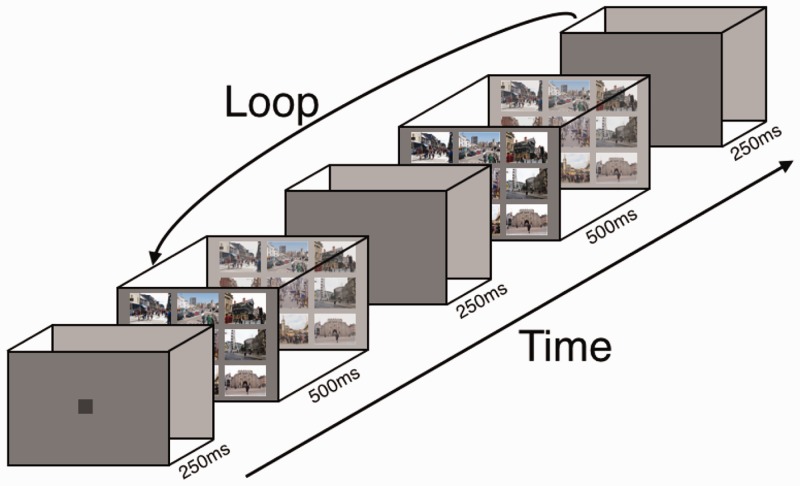
Example of modified flicker paradigm stimulus in nonaplex format (adapted from Rensink et al., 1997).

Participants viewed 27 trials containing one, four or nine scenes (with 9 trials of each type of display). The experiment used a modified flicker paradigm (see Figure 3). The original target image and accompanying foil images (for all multiple scene versions) were displayed for 500 milliseconds, followed by a blank lasting 250 milliseconds. The display then showed the modified target image and foils for 500 milliseconds, followed by a second blank of 250 milliseconds. This sequence continued in a loop either until correct detection was made (indicated by a button press, and the participant verbally reporting the changed item and clicking on the scene that contained the change in the multiplex versions), or until the cut-off point of 180 alternations (four and a half minutes). This cut-off point was chosen following pilot work; this allowed most of the changes to be detected, even for nonaplex displays, but avoided the demotivating and frustrating situation for participants when changes were not detected for very long periods (as prior piloting found that some changes in nonaplexes could go undetected for over 5 minutes viewing). Trials were presented in a random order, interleaved across conditions. Between participants, stimuli were counterbalanced across the three conditions such that each scene appeared equally in each of the monoplex, quadraplex and nonaplex displays.

#### Analysis

Data in these experiments were analysed using Cox Regression survival analysis, a technique that has previously been used for event history style examination of data (Hirose, Kennedy, & Tatler, 2010; Pannasch, Dornhoefer, Unema, & Velichkovsky, 2001). This method uses observed performance to plot the likelihood of a change being detected at any given instance in time during the trial (given that the participant has not previously detected the change).

During piloting for this study, participants often struggled to detect changes (particularly in nonaplex displays), thus prompting the addition of a set time-out point of four and a half minutes for each trial. By using survival analysis it is possible to take into account trials where participants were unable to detect the change (i.e. in a medical experiment these would be trials in which participants have ‘survived’ the trial). Survival analysis also provides a change detection probability at the end of the trial time limit that represents the overall performance (how many trials were successfully detected). Thus the analysis incorporates aspects of both time and accuracy in the task, which is preferable to simply removing the trials where participants were unable to detect the changes within the given time limits. For this use of survival analysis, the assumption is made that, given an unlimited amount of time participants will at some point detect the change in all trials across all conditions. Here the figures presented are inverse survival functions of the data. The method means is it possible to effectively plot the probability that a change will be detected at any given time point (given that it has not previously been detected). This presentation of the data is more readily interpretable than plotting the survival functions themselves, which plot the probability that the change will not be detected at any given point in time, and is similar to interpret to cumulative frequency curves.

Data were analysed using the survival (Therneau & Grambsch, 2000) package in R. We report Wald statistics, which quantify whether the survival model parameters are better fit when a factor of interest - such as (in this case) the number of scenes - is included. The statistical comparison tests for the degree of overlap between survival functions, with the null hypothesis being that the functions are superimposed. As Wald values increase, this represents an increasing difference in performance between conditions. For pairwise comparisons between levels, we report *z* scores. All participants' data are included in each model, with the individual variance accounted for by using the cluster function. This means, that each participant is represented multiple times in the data - but as each participant completed the same number of trials across each condition in each experiment, that all participants are represented equally in the analysis.

### Results

**Figure 4.**
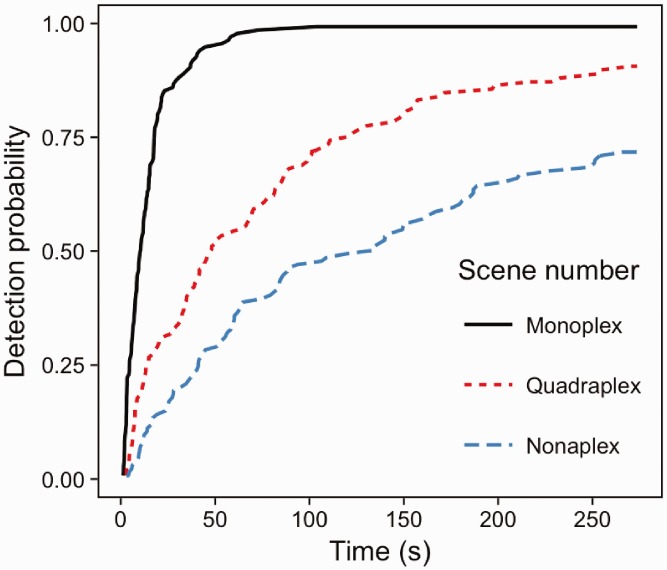
Inverse survival plots for monoplex, quadraplex and nonaplex change detection.

A strong, significant association of scene number with detection speed and accuracy was found (Wald (2) = 323.3, *p* < .001), with participants detecting changes more rapidly in single scene viewing than quadraplex and nonaplex versions of the task. Figure 4 demonstrates that by 100 s after the onset of the flicker display, changes in single scenes were almost at 100% probability of being detected (i.e. the probability of detection is close to 1). At the same point in time, the probability of detecting a change within a quadraplex display was about 0.7, and even lower at about 0.5 for nonaplex scene viewing. The levels of scene number (monoplex, quadraplex and nonaplex) were compared using Forward Difference coding (mono vs quad, quad vs nona). There was a significant difference in detection performance between monoplex and quadraplex displays (*z* = 10.26, *p* < .001) and between quadraplex and nonaplex displays (*z* = 11.93, *p* < .001).

### Discussion

Experiment 1 examined change detection in monoplex, quadraplex and nonaplex displays to establish the constraints on detection performance when searching for a change in a pictorial array when the nature of the visual change was unknown, and the number of distracting scenes was varied systematically. The time taken to detect changes in monoplex displays was similar to that reported in previous change detection studies using the flicker paradigm (such as Rensink et al., 1997). In quadraplex displays not all changes were detected within four and half minutes. Final accuracy fell from approximately 99% for monoplex to ∼92% for quadraplex. Accuracy was further reduced for nonaplex displays, with only ∼73% of changes detected within 180 alternations between the modified and unmodified versions of the display.

One explanation for multiplex detection difficulties is the increase in the physical extent of the visual array when adding multiple scenes, however, increasing the number of scenes in a multiplex array also necessarily increases the total amount of information in the display. Furthermore, multiplexes introduce physical separation between scenes. All of these factors (physical extent of the search array, information content of the display and physical separation) may introduce behavioural costs for change detection.

## Experiment 2

Experiment 2 examined three possible sources of multiplex detection difficulty based on the consequences of presenting multiple scenes as opposed to presenting one scene. Firstly, adding scenes (while maintaining image size ratio) increases the physical visual angle over which information is displayed. Secondly, an increase in the amount of visual information compared to a scene shown on its own increases the number of possible change locations. The third possible cause is that the difficulty is not due to the visual content, but that performance is driven by the segmentation of a multiplex display into a set of physically separated ‘windows’, as is typically found for multiplex displays.

Physically larger arrays usually lead to longer search times (Carrasco & Chang, 1995), however, the inherent structure of real-world scenes may reduce the effect of the increased physical display size (Ball & North, 2005). To examine whether the total physical area occupied by the images influences change detection, change detection performance was compared when a scene was displayed at 400 × 300 pixels to performance when displayed at 800 × 600 pixels. This allowed control of the presentation of identical visual content, but manipulates the total physical area over which observers must search for the change.

To manipulate the influence of visual information content in displays, participants were presented with one quadrant of the original scene (the quadrant containing the change) at 400 × 300 pixels and performance was compared in this condition to performance when the entire scene was displayed at the same size (400 × 300 pixels). Thus the physical extent of the image was constant, but the information content differed between these two conditions.

To assess the influence of the physical segmentation of a multiplex display (with each scene presented in a separate ‘window’) change detection performance when a scene was presented in 800 × 600 pixel format was compared with performance when the same scene was presented, but split into four 400 × 300 quadrants and separated by 50 pixel spaces. This manipulation allowed us to consider whether multiplex displays are difficult to process for change detection because of the separation of information into separate panels. Such discontinuity may, for example, result in the viewer parsing the display into a set of separate scenes, dealing with each as a separate perceptual ‘unit’. If viewing information in this way disrupts people's ability to detect changes it would suggest that the spatial arrangement, as opposed to the visual content, of the multiplex display impacts upon attentional allocation in multiplex arrays. In Stainer et al. (2013a), we found only a very minimal (and not significant) change in viewing behaviour when scenes were split into quadrants and moved 50 pixels apart. Varakin and Levin (2008) found no effect of windowing on change detection performance, however, given the aims of the previous research, the window frame was overlaid on top of the scene, whereas here we moved the content apart to examine whether physical separation (in a condition with no loss of content, to examine whether detection performance was affected.

**Figure 5.**
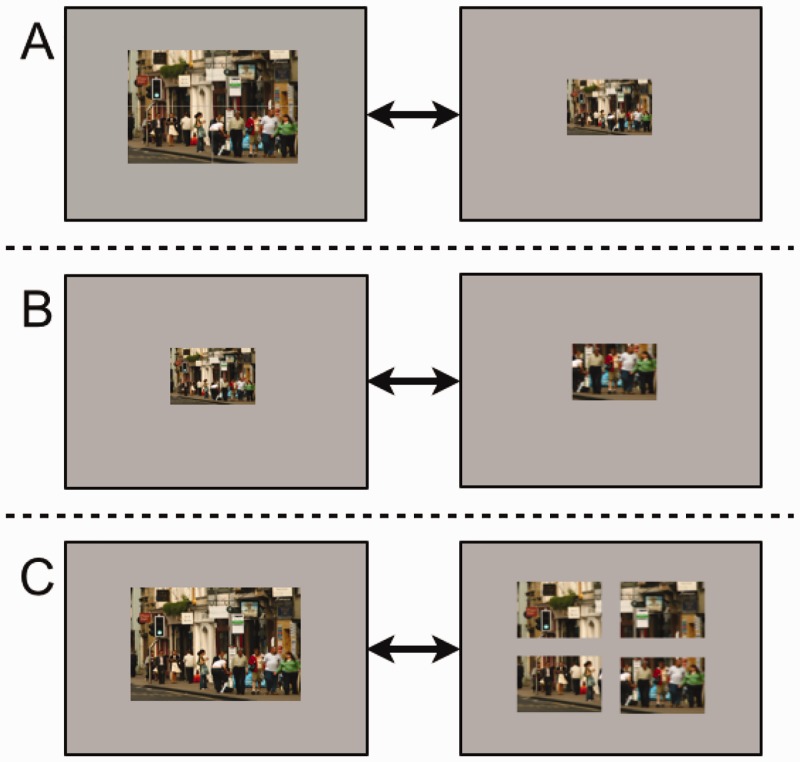
Summary of the main experimental hypotheses for Experiment 2 using examples from the experimental image set. (A) Large scene versus small scene conditions where visual content is controlled, and physical display size is manipulated. (B) Small scene versus quadrant condition where physical display size is controlled and the amount of visual content is manipulated. (C) Large scene versus separated scene conditions where visual content and size are controlled, but the number of artificial ‘scene divisions’ is manipulated.

The three main experimental comparisons of Experiment 2 are illustrated in Figure 5, addressing three questions. Firstly, to what extent does the physical size of a display influence change detection? Secondly, can increasing the amount of information in a display account for the difficulties in detecting changes across multiple scenes? Finally, does the division of a scene into four scene sections create the same difficulties as moving from a monoplex to a quadraplex display?

### Methods

#### Participants

Fifteen students participated in exchange for course credit. All participants had normal or corrected-to-normal vision. None had participated in Experiment 1.

#### Stimuli

Thirty-two photographs of city centre scenes were used. Photographs were scaled to 800 × 600 pixels in size and prepared in four ways. They were either presented whole (large), split into four quadrants (separated) and presented with an inter-image gap of 50 pixels horizontally and vertically, or scaled by 50% to 400 × 300 pixels (small). In the fourth condition only the changing quadrant of the large scene version (400 × 300 pixels in size) was used for presentation. Examples of all display types can be seen in Figure 5.

#### Procedure

The task of the participant was to detect a change in a flickering image or image array. Participant viewed a total of 32 trials, with eight trials of each display condition. Experimental presentation times were the same as the previous experiments, with the experiment cut-off time set to 180 seconds.

### Results

In the quadrant condition, change detection was at 100% with all changes being detected by the cut-off point. Detection rates were lower for small, large and large exploded display conditions (92.5%, 91.7% and 91.7% respectively) with no observable differences between these conditions.

**Figure 6.**
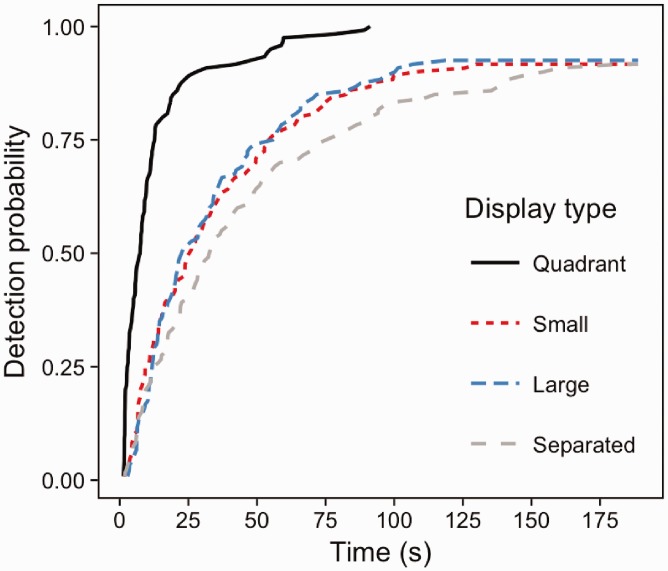
Inverse survival functions for the four different display conditions in Experiment 2. Note that quadrant detection probability reaches 100% at 91.22 seconds.

Analysis showed a significant association between display condition and detection performance (Wald (3) = 98.92, *p* < .001; Figure 6), though this effect was strongly driven by the single quadrant display condition. We examined the differences between conditions by changing the reference condition in the model. Quadrant change detection was indeed significantly better than performance in large (*z* =−7.84, *p* < .001), separated (*z* =−9.5, *p* < .001) and small (*z* =−7.85, *p* < .001) displays. There were no differences between the large, small and separated conditions (all *p*-values > .05).

### Discussion

The data showed no significant difference in change detection between the large version of the scene and the small version of the scene. When the same visual information was presented on an area four times larger, change detection was unaffected. While physical display increases may require longer saccade lengths to navigate, the bounds of scene structure, in terms of the consistent spatial relationship between the items in the images in each of these conditions may compensate for the scaling of the image. Similar scaling mechanisms to counteract these physical issues have been proposed in reading (Morrison & Rayner, 1981). The result of Experiment 2 demonstrates that increasing physical display size cannot account for the increased change detection times at multiple-scene viewing from Experiment 1.

Participants took less time, and detected more changes when presented with a quarter of the image, compared to the whole image presented at the same dimensions. This result suggests that increasing the amount of visual content in a multiplex display influences the difficulty of detecting changes. This suggestion is consistent with studies finding that change detection times increased when the number of items (and therefore information content) in an array is increased (Bravo & Farid, 2004; Rensink, 2000; Wilken & Ma, 2004) and also when the number of items in a single scene is increased (Beck et al., 2007). This result is entirely consistent with Yokosawa and Mitsumatsu (2003), who found that change detection performance from scenes that were scrambled and had various number of segments revealed, was related to the number of segments, rather than the coherence of the scene.

While performance was influenced by the information content of the scenes, it was not influenced by whether or not the scene was physically separated into four separate windows in the display: there was no difference between the small and the large-exploded version of the scenes, where both have the same visual content, but are arranged to appear like the monoplex and quadraplex versions of Experiment 1. Thus the reduction in change detection performance found in quadraplex arrays was not principally a result of the division into multiple scenes. This corresponds with our previous findings (Stainer et al., 2013a) and the findings of Varakin and Levin Varakin and Levin (2008; also see Bi et al., 2010; Hutchings et al., 2004; Tan & Czerwinski, 2003; Yokosawa & Mitsumatsu, 2003), where change detection was not influenced when images were presented in windows.

## Experiment 3

When participants view a single scene, they only have to process one scene gist. It may be that the demands of processing several scene gists are analogous to findings from visual search (such as Treisman & Gelade, 1980), where set-size effects are moderated by the similarity of stimuli. If this provides an explanation of the difficulty that participants experience in multiple-scene change detection, then introducing differences in context between scenes should further increase the difficulty of detecting changes (Beck & Ambler, 1973). Alternatively, if the variability between the content across scenes does not matter, we would expect that increasing differences in context should not influence change detection performance. To explore this point, in Experiment 3 the similarity of pictorial context between scenes within a multiplex array is manipulated.

To investigate the nature of the semantic relationship between scenes, changing scenes were presented in a quadraplex array of scenes that were scene-consistent (i.e. the additional scenes would make up one large picture), scenes that were similar to the target scene (i.e. all scenes were of the same semantic category, such as city centres), or scenes that were all of different types (i.e. drawn from different semantic categories: Motorways, airports, car parks and city centres). Scene categories were selected to contain similar items, and similar focal lengths, from locations familiar to the participant group lifestyles but from well-defined easily discriminable categories. While visual search is influenced by item similarity (both between target and distractors, and in the distractor set), scene gist has yet to be explored as a factor in which similarity may affect target search tasks.

### Methods

#### Participants

Fifteen undergraduate students participated in exchange for course credit. Participants had not taken part in either previous experiment.

#### Stimuli

144 photographs were collected; 36 of each of the four different scene types; city centre, airport terminal, car park and motorway scenes. Photographs were 800 × 600 pixels in size, and split into four 400 × 300 pixel quadrants. In each of the sets of 36 images, 12 were randomly selected to be targets. In the target images, changes were made equally often to each of the four quadrants across the image set. Non-target images were split into four quadrants, with experimental distractor items being selected equally from the four quadrants.

**Figure 7.**
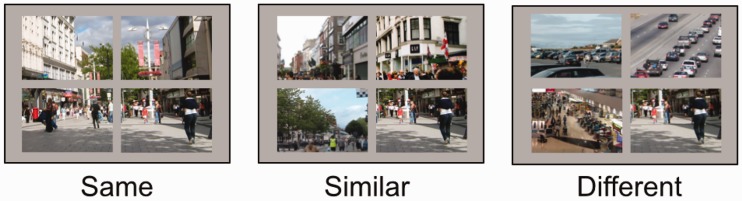
Examples of displays where the windows are from the same scene, semantically similar scenes (e.g. all city centre) and semantically different scenes.

Stimuli were presented in quadraplex displays in all trials (see Figure 7). The similarity between the target and distractor scenes was varied across three experimental conditions: The three distractor (i.e. unchanging) quadrants were (1) drawn from the same scene, (2) similar scenes (drawn from the same semantic category as the target), or (3) different scenes (drawn from different semantic categories to the target scene). Presentation order was randomised across trials and the non-changing distractor images were selected randomly for each trial. A pilot study [*n* = 5] ensured that when presented in the image-consistent format, detection was made in fewer than 40 alternations in over 50% of the trials (a level of performance for quadraplex displays that is in line with that found in Experiment 1).

#### Procedure

Participants viewed 48 trials, with 16 trials of each different condition. In each trial the participants were required to report which of the four quadrants had contained the changing object. The trial cut-off time was set to 120 seconds.

### Results

**Figure 8.**
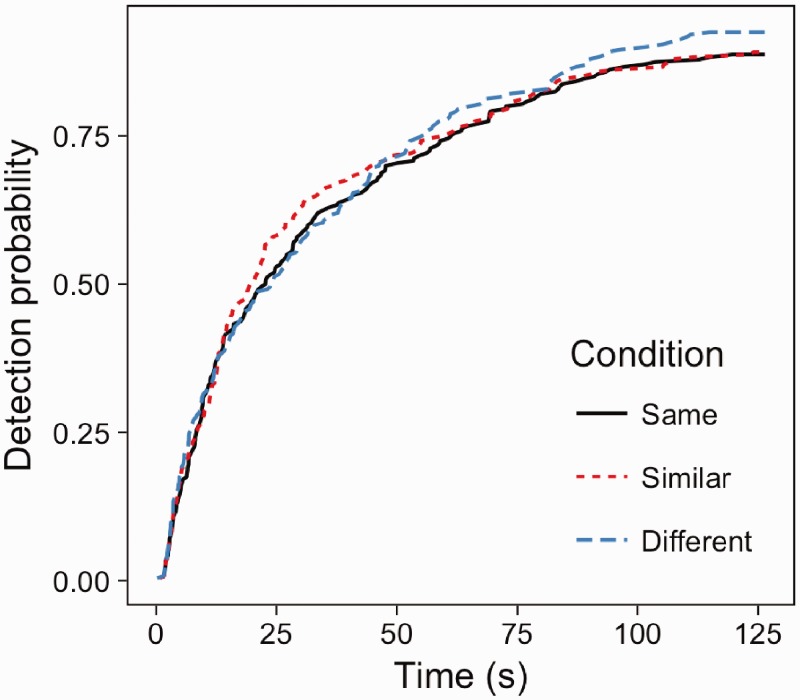
Examples of displays where the windows are from the same scene, semantically similar scenes (e.g. all city centre) and semantically different scenes.

Cox Regression analysis revealed no significant association between distractor-scene similarity on detection probability (Wald (2) = 0.9, *p* = .64; Figure 8). Changes were detected at an equal speed and accuracy in scene-consistent, similar and different scene conditions.

### Discussion

While caution must always be drawn from interpreting null effects, survival functions for single item change detection in quadraplex displays were inseparable using statistical tests, regardless of the similarity between the scene gist of the target scene and the three distractor-scenes. Of course at some level adding scenes to a display must necessarily increase the variation in semantics across the display: Even images (quadrants) drawn from the same overall scene will each have slight variations in semantic content. However, the findings from Experiment 3 suggest that the accuracy and response latency for detecting changes in quadraplex arrays of photographic scenes are not necessarily influenced by the amount of semantic similarity between the target scene and the three distractors. Thus, even when semantic variation is high - in the case where distractor scenes are each drawn from a different category and are all from different categories to the target scene - change detection can proceed in the same manner as when the semantic variation and separation between scenes is very low - when all images are taken from the same overall scene.

These findings contrast with the influence of semantic similarity on search performance in more simple displays. When searching for a target object among distractors search performance is influenced by the semantic similarity between the target and distractors (Jonides & Gleitman, 1972). However, when searching for a change within a single scene of a multiplex display, the results of Experiment 3 suggest that semantic consistency between scenes did not influence performance. One potential consideration is that it is difficult to separate the semantic, and visual similarity of scenes. We did not select scenes with the intent of investigating visual similarity, and we cannot therefore rule out the possibility that there may be confounds between these two factors.

One possible cause of this pattern of results is that the reason scene gist did not appear to be important is that knowledge of scene gist gave no advantage to the detection task. The change could have occurred in any of the scenes and was not linked to scene semantics, therefore making scene gist less informative. In tasks where scene gist is more task-relevant or diagnostic of the likely change (such as ‘find the car’, making traffic scenes more likely than airport terminals to contain a target), the competition between gists may still be more analogous to classic visual search findings.

The results from Experiments 1 to 3 seem to indicate that the factors that govern attention allocation in the multiplex might be the same as those that have been shown to have influence in single scene viewing. This would suggest that people might be treating the multiplex as though it were one scene: i.e. using the whole multiplex as the frame of reference for viewing. This finding is at odds with our previous work where we demonstrated that when a single scene was scrambled to form a multiplex, fixations were allocated to each quadrant as though each were a single scene (an individual scene-based frame of reference Stainer et al., 2013a). This apparent contradiction might be due to the task of the observer. If observers are able to flexibly segment these displays depending on the demands of the task, we could expect that the frame of reference for viewing would determine the factors that influence attention allocation. One way to examine this is to ask what happens when we reintroduce the importance of the individual scene-level frame of reference, which we did in Experiment 4.

## Experiment 4

In Experiment 4, we gave the participants prior knowledge of which scene the changing target would appear in. Prior knowledge about the spatial likelihood of targets has been shown to influence the way people search for targets (Torralba et al., 2006; Zelinsky & Schmidt, 2009). In previous change detection experiments, cuing the location of the change by use of a gaze cue was found to reduce the deleterious effects of additional pictorial information (Langton, O'Donnell, Riby, & Ballantyne, 2006; O'Donnell & Langton, 2003). This finding is consistent with the notion of a narrowing of the search set to relevant locations in the scene. Similar narrowing of the search space has been found when searching for objects in natural images. Zelinsky and Schmidt (2009) asked participants to search for UFO targets in aerial-view maps. They found that cueing a target's location effectively constrains participants' visual search to only the cued locations. Previously, various forms of cues have been shown to successfully improve the detection of changes in natural images. Scott-Brown and Orbach (1998) demonstrated that cueing the location of a changed item in an array of contrast patches significantly reduces the threshold of change in contrast required for successful detection. This experiment also provided evidence that cueing reduces the set-size effect in a change detection task, similar to the findings of cueing subsets of items in visual search (Palmer, 1994; Palmer, Ames, & Lindsey, 1993).

If the observer uses a multiplex-based frame of reference for searching for the change, we would expect that the content of the distractor scenes alone (task relevant or not) should influence attention allocation. In contrast, if performance is improved by cueing, this would suggest that the frame of reference is flexibly fitted to the demands of the task, and that task irrelevant content (i.e. the distractor scenes) should not affect detection times.

### Method

#### Participants

Twelve psychology undergraduate students took part in this experiment. All completed the experiment for course credit and had not completed Experiments 1 to 3.

#### Stimuli

Experiment 4 used the same 126 digital colour photographs of city centres as used in Experiment 1.

#### Procedure

The procedure of Experiment 4 was identical to Experiment 1, except that in half of the trials, the location of the changed scene in the multiplex array was cued before each trial for 500 ms by means of a dark grey rectangular cue. The scenes that were provided a cue were counterbalanced across the experiment so that all images were presented equally in the cued and un-cued condition.

### Results

**Figure 9.**
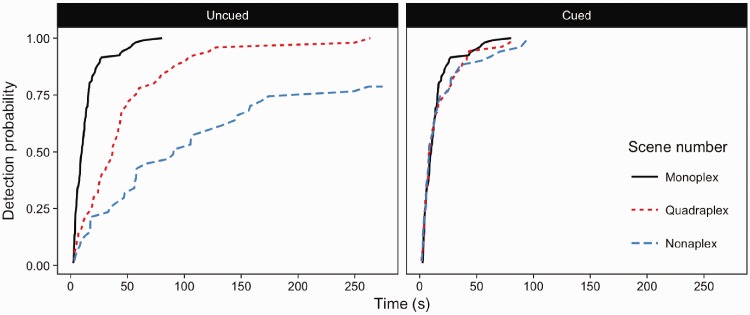
Inverse survival functions for monoplex, quadraplex and nonaplex detection trials in un-cued (upper panel) and cued (lower panel) conditions.

In the cued condition, changes were detected 100% of the time before the cut-off in all scene number conditions. In the un-cued conditions performance was comparable to Experiment 1 (Figure 9).

There was a strong relationship between scene number and detection speed in the un-cued condition (Wald (3) = 160.3, *p* < .001), showing the same pattern of results as observed in Experiment 1. Monoplex was faster than quadraplex (*z* =−7.028, *p* < .001), with nonaplex version taking longer still (*z* =−12.103, *p* < .001). In the cued condition, no such pattern was observed (Wald (2) = 1.27, *p* = .53), with changes being detected at the same performance in all conditions (all *p*-values > .05).

**Figure 10.**
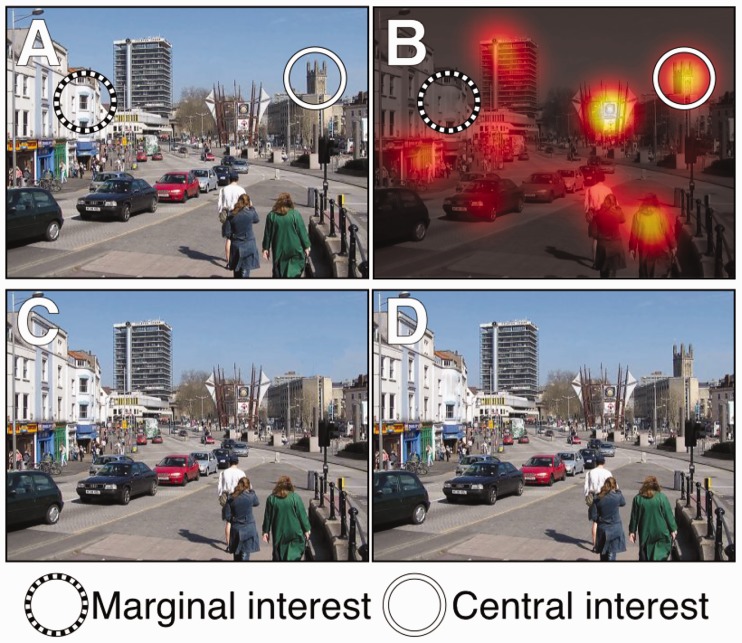
Examples of the original image (a), a ‘heat-map’ of the selected locations of interest for the image by the 8 observers (b), and the changed central-interest (c) and marginal-interest (d) versions of the images.

In monoplex trials, participants detected the change within 75 s, and typically all changes were detected (Figure 10, upper panel). In the quadraplex format of the un-cued condition, detection accuracy after 75 s is over 20% lower, with the nonaplex detection being significantly slower. However, in the cued condition, performance was the same across all display conditions (Figure 10, lower panel).

### Discussion

Providing a cue for the location of the scene containing a change within a multiplex display simplified the change detection task to the extent that survival functions for change detection in monoplex, quadraplex and nonaplex were essentially overlapping. This finding contrasts with the widely separated survival functions seen in un-cued conditions, where increasing scenes in the array were associated with increasing detection latencies and reduced detection accuracy. When the target scene in a four or nine scene multiplex was known, the additional irrelevant scenes in the array did not affect detection performance. This finding provides good evidence that attentional cueing negates the effect of increased visual load of the multiple-scene display conditions by limiting the area over which participants need to attend. Further, it demonstrates that although in some viewing conditions observers might use multiplex-based frames of reference (Experiments 1 to 3), when beneficial to the task observers might switch to an individual scene-based frame of reference. Importantly this means that the distractor scenes do not appear to affect attention allocation when irrelevant to the task being conducted. Given that there are times when we know, or do not where the task relevant information will be in a display, understanding how people cope with both types of task will be important to understand how people attend to multiplexes in various viewing conditions.

## Experiment 5

Experiment 4 suggested that when target location is known, change detection performance was unaffected by being presented in a multiplex. Another feature that might affect change detection performance is that of the target itself, in particular its importance in the scene. Rensink et al. (1997) showed that changes to items in a scene that were mentioned in descriptions by at least three of six judges when asked to describe ‘what the picture was about’ were detected significantly faster than items that were not mentioned. This central versus marginal interest comparison somewhat mirrors the earlier findings of Yarbus (1967) and Buswell (1935) who showed that we prioritise information that is interesting when inspecting pictures.

When completing tasks across multiplexes, it is likely that some information is more interesting, or important than others. It is unclear what effect multiplexing might have on central and marginal interest changes, and whether detection performance will therefore change in the same way for both types of changes as we increase the scene numbers. Experiment 5 sought to examine how such changes are detected across multiplex displays.

### Methods

#### Participants

Twenty-four students took part in Experiment 5. All participants had not taken part in any other experiment.

#### Stimuli

The stimuli used in Experiment 5 were the same as Experiment 1, except that new changes were made to the images. Each image had two changed versions created. As the pictures contained less basic narrative than the original Rensink et al. (1997), we used a slightly different approach to selecting marginal and central interest items in the images. 8 observers naive to the study were asked to click the 5 most “interesting locations” in the pictures. Participants were simply told to select the 5 most interesting locations�. The instructions meant that participants could select objects that they thought were semantically interesting, or areas that were perceptually interesting. We selected the central interest items as items in the photographs that least 6 out of the 8 observers, with marginal interest items being selected as objects that were clicked on by none. Examples of the selection process, and example changes can be seen in Figure 10.

#### Procedure

The procedure of Experiment 5 was identical to Experiment 1. Marginal and central interest changes were counterbalanced across the experiment such that each participant saw trials of central and marginal interest changes, and each scene occurred equally across scene numbers and change type. Participants were not told of the marginal-central change structure until after the experiment when they were fully debriefed.

### Results

**Figure 11.**
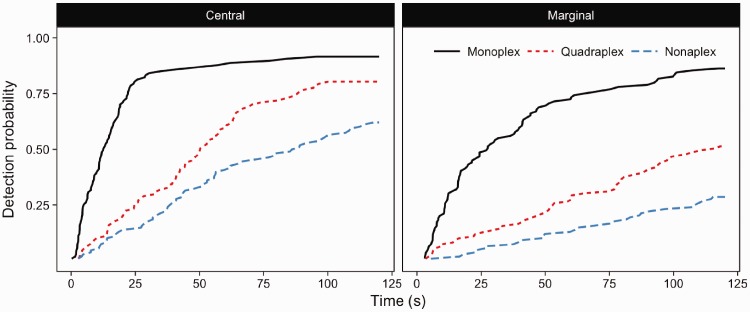
Survival plots for central versus marginal interest changes.

**Figure 12.**
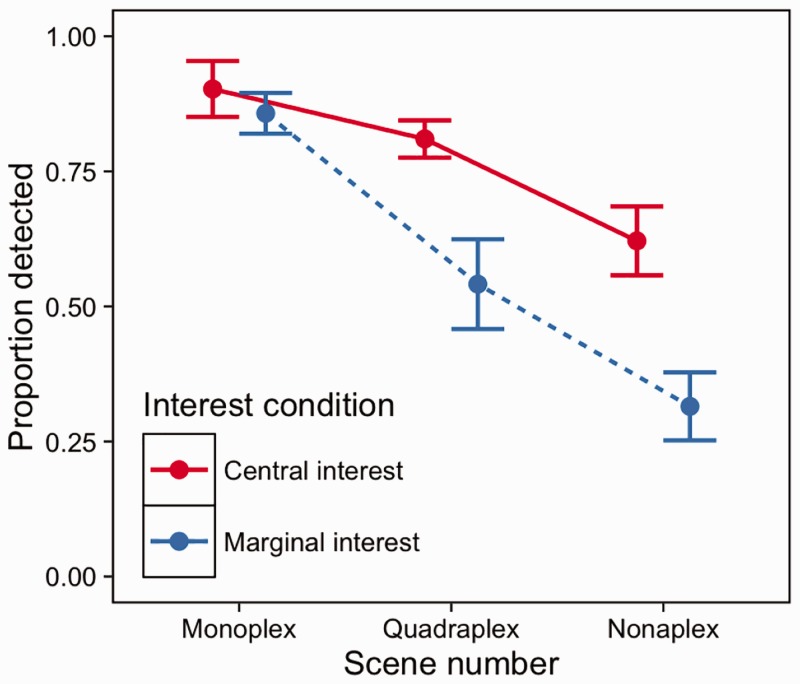
Mean proportion of changes detected across observers in the two conditions with 1 ± SE bars.

There was a significant effect of scene number and interest condition on detection performance (Wald (3) = 106.7, *p* < .001; Figure 11). Detection became more difficult across increasing scene number (*p*-values < .001), and there was a significant difference between marginal and central interest changes (*z* =−6.105, *p* < .001). We used an ANOVA to compare overall proportion of changes that were detected by the cut-off point to examine whether interest and scene number interacted. Figure 12 shows that on overall proportion of changes detected, there was a significant interaction between scene number and interest condition (*F* (2,38) = 6.268, *p* = .004); increasing scene number had a larger effect on marginal interest changes than central interest changes.

### Discussion

Multiplexing made both central and marginal interest changes more difficult to detect - but difficulty did not increase at the same rate. The difference between performance in central and marginal interest changes increased with scene number, suggesting that detection of marginal-interest changes suffered more from increasing scene number than detection of central-interest changes. This finding is in line with previous reports of semantic interest being linked to change detection performance (O'Regan, Rensink, & Clark, 1999; Rensink et al., 1997), but indicates that with increasing scene number - the difference in performance increases. This finding has important implications for multiplex tasks, where it demonstrates that information that is not important to the task being currently undertaken may be progressively hidden as the multiplex size increases. While previous reports (Tickner & Poulton, 1973), and the results here have shown that detection becomes more difficult across multiplexes, we demonstrate here that semantic interest can modulate the degree to which performance is affected.

## General discussion

The need to assess human perceptual limits objectively when evaluating display technology is particularly underlined by recent research on visual perception that demonstrates not only that our visual awareness may be quite limited, but also that people systematically overestimate their ability to process visual information (Beck et al., 2007; Levin, Momen, Drivdahl, & Simons, 2000). Previous research on multiplex viewing has tended to focus on how detection of events is related the number of screens in multiplexes (e.g. Tickner & Poulton, 1973), but until now there has not been an exploration of the sources of this processing difficulty. In a series of experiments, we explored several potential candidates for the problems associated with attention allocation when attending to multiplex displays.

Experiment 1 found that change detection performance became worse as the number of scenes increases in a multiplex. The findings from Experiment 2 showed that there was no significant effect of the contributions of increased physical display area, or whether a scene was displayed intact or segregated into four separate windowed displays (mimicking a quadraplex display). However, there was a clear effect of the total amount of visual information in the display, with more visual information being associated with longer detection latencies. In Experiment 3, the between-scene semantic gist variability between scenes could also not account for the difficulties of multiplex change detection. One interpretation of the findings in these three experiments is that despite the challenges associated with attending to a multiplex that are not present when attending to a single scene, how people allocate attention might be dependent on many of the same factors that influence single scene perception. This is somewhat surprising, given the physical and semantic separations and variation between scenes in a multiplex, factors that have been shown to affect performance in tasks when this variance exists between objects in an array (Beck & Ambler, 1973; Treisman & Gelade, 1980). Attention allocation here did not appear to be disrupted by the need to cross physical and semantic boundaries in multiplex displays. Neither was the variation in gist problematic for scene processing: there was no evidence for costs associated with increased semantic variation across scenes. It would appear that of these possible sources of difficulty, only the amount of information across the entire display had a significant impact on how attention was allocated (particularly supporting the findings of Yokosawa and Mitsumatsu (2003)). These findings taken together suggest that observers used the multiplex itself, rather than the components of the ‘scene units’, as the frame of reference for inspection. This is somewhat different from the findings of our previous research (Stainer et al., 2013a), where we found that fixation behaviour in scrambled scenes (i.e. multiplexes with discontinuity between parts) was driven towards the centre of the scene units, albeit with an early tendency to fixate centrally in the multiplex.

In Experiment 4, we examined whether the reason that people appeared to use the multiplex as a frame of reference might that in the change detection task studied, scene bounds provided no task-relevant information (i.e. they did not give clues to where the target was). We reintroduced the importance of the scene bounds by only cueing one of the scenes in the multiplex. This meant that if observers were unable to change frames of reference from the entire multiplex, to an individual scene based frame, then performance should be identical to un-cued trials. This would suggest that the additional information itself should cause additional processing difficulty. Given that in multiplex viewing, we might have an expectancy of where to find the target of interest, scene bounds would be useful to the task, and therefore we would expect that participants would likely only search the task-relevant scene and be unaffected by the other information. We found support for the latter of these two possibilities: observers performed in multiplex cued trials as though there was only one scene in the display.

Previously, we observed monitoring behaviour in a real, complex multiplex CCTV environment (Stainer et al., 2013b). In this study, we found that operators preferred to seek out criminal activity on their single scene monoplex display, demonstrating some of the guidance that may be occurring in Experiment 4. By reducing the set-size of a display to locations informed by prior-knowledge (e.g. ‘Where is a crime likely to appear at this time of day?’), multiplex-users are likely seeking to make the task more manageable, effectively turning their multiplex into a monoplex.

Experiment 5 demonstrated that the manner in which change detection performance was modulated by scene number depended on whether the change was semantically important, with an increasing difference between central and marginal interest changes as scene number increased. These findings have clear implications for tasks that occur across multiplex displays, with prior knowledge of where the target is likely to be, and whether the target is semantically relevant (which it is likely to be so for the task being conducted) both negating the performance costs of multiplexing. This, of course, comes at the detriment of targets that are not important to the task that is currently being conducted. In many multiplex monitoring tasks, unexpected events that occur in unexpected locations are no less important to the task than expected events in expected locations.

What can we generalise from our change detection task to other tasks in the multiplex? The reason for using change detection here was to provide a task where the target's location could be anywhere in the scene (it was not cued by scene context as a ‘find the car?’ task would be - cueing road areas), and anywhere in the display. There are many tasks conducted on multiplex displays that share similarities with the change detection task. For example, detection of an unattended bag in an airport scene that involves noticing an object that is not in the ‘unchanged’ version of the scene. It should be borne in mind that in security scanning, the set of possible targets is unknown and unspecified, it is not a general search for an individual or a specific item. However, there are many tasks that involve much more guided searching, such as searching for a face in a crowd. Here, we show that when not all information is relevant to the task, performance seems more related to the subset of cued information. Thus, guidance from target-scene contextual relationships will likely go some way to making multiplex tasks less complex. Nevertheless, in difficult multi-screen search tasks, we can predict a similar pattern of results as we report here, where the amount of information critically influences response latency, and scene guidance can reduce search times.

### Conclusions

Many of the tasks that are conducted across multiplex displays rely on detecting events that occur somewhere in the display. Here, we provide the first systematic evaluation of factors that make multiplex detection difficult. Information load appears to be the dominant factor in detection performance, with more information leading to worse performance. It did not matter whether this was from one scene, or many different scenes. If only a subset of the information was important for the task, observers adjusted their ‘set-size’ to show improved performance (as in Zelinsky & Schmidt, 2009). If the target itself was important to the scene, changes were preferentially detected compared to targets that were not important, with the magnitude of difference increasing with scene number. These findings have important implications for considering the task of human observers across multiplex displays, particularly given that the overriding message from the set of experiments presented here is that detection performance in a multiplex is not as solely driven by the number of scenes in a display.
